# Statistical power in parallel group point exposure studies with time-to-event outcomes: an empirical comparison of the performance of randomized controlled trials and the inverse probability of treatment weighting (IPTW) approach

**DOI:** 10.1186/s12874-015-0081-3

**Published:** 2015-10-15

**Authors:** Peter C. Austin, Tibor Schuster, Robert W. Platt

**Affiliations:** Institute for Clinical Evaluative Sciences, G106, 2075 Bayview Avenue, M4N 3M5 Toronto, ON Canada; Institute of Health Management, Policy and Evaluation, University of Toronto, Toronto, Canada; Schulich Heart Research Program, Sunnybrook Research Institute, Toronto, Canada; Clinical Epidemiology and Biostatistics Unit and Melbourne Children’s Trial Centre, Murdoch Children’s Research Institute, Royal Children’s Hospital, Parkville, VIC Australia; Department of Epidemiology, Biostatistics and Occupational Health, McGill University, Montreal, Canada; Department of Paediatrics, University of Melbourne, Melbourne, Australia; Department of Pediatrics, McGill University, Montreal, Canada

**Keywords:** Observational study, Propensity score, Inverse probability of treatment weighting, Causal inference, Survival analysis, Randomized controlled trial, Monte Carlo simulations

## Abstract

**Background:**

Estimating statistical power is an important component of the design of both randomized controlled trials (RCTs) and observational studies. Methods for estimating statistical power in RCTs have been well described and can be implemented simply. In observational studies, statistical methods must be used to remove the effects of confounding that can occur due to non-random treatment assignment. Inverse probability of treatment weighting (IPTW) using the propensity score is an attractive method for estimating the effects of treatment using observational data. However, sample size and power calculations have not been adequately described for these methods.

**Methods:**

We used an extensive series of Monte Carlo simulations to compare the statistical power of an IPTW analysis of an observational study with time-to-event outcomes with that of an analysis of a similarly-structured RCT. We examined the impact of four factors on the statistical power function: number of observed events, prevalence of treatment, the marginal hazard ratio, and the strength of the treatment-selection process.

**Results:**

We found that, on average, an IPTW analysis had lower statistical power compared to an analysis of a similarly-structured RCT. The difference in statistical power increased as the magnitude of the treatment-selection model increased.

**Conclusions:**

The statistical power of an IPTW analysis tended to be lower than the statistical power of a similarly-structured RCT.

## Background

Randomized controlled trials (RCTs) are considered the gold-standard for estimating the effects of treatments, interventions, and exposures. The primary advantage of well-designed and conducted RCTs is that they result in unbiased estimation of treatment effects, since treatment assignment is not confounded with patient prognosis. However, there is an increasing interest in using non-randomized or observational data to estimate these effects.

There are multiple reasons for the increasing interest in using observational data to estimate the effects of treatments, interventions, and exposures. First, due to the use of restrictive inclusion and exclusion criteria, patients included in some RCTs may not be reflective of the patient population in which the treatment or intervention will ultimately be used. In contrast to this, observational studies permit the estimation of treatment effects in patient populations reflective of those in which the treatment is currently applied. Second, the comprehensive care and thorough follow-up provided to patients in some RCTs may not be reflective of the standard of care that is provided to patients outside of the tightly controlled confines of an RCT. In contrast, observational studies permit the estimation of treatment effects in settings reflective of how the interventions and treatments are used in current practice. Third, observational studies permit the estimation of the effects of interventions for which it would be unethical to randomize patients to treatment (e.g. smoking or radon exposure). Fourth, the growing number of large health care databases providing population coverage at the state, provincial, or national level permit observational studies to be conducted relatively quickly and inexpensively compared with the time and cost required to conduct a comparable RCT. The primary limitation of observational studies is that treatment allocation can be confounded with patient characteristics: treated patients often differ systematically from control patients. Failure to account for this confounding will result in biased estimates of treatment effects.

Statistical methods to account for confounding in observational studies are essential to obtaining unbiased estimates of treatment effects. There is increasing interest in using propensity score methods to reduce or minimize the effects of confounding due to measured covariates when using non-randomized data to estimate the effects of treatments and interventions. The propensity score is the probability of receiving the treatment conditional on measured baseline covariates [1–3]. There are four different methods in which the propensity score can be used to minimize the effects of measured confounding: covariate adjustment using the propensity score, stratification on the propensity score, matching on the propensity score, and inverse probability of treatment weighting (IPTW) using the propensity score [1–5]. The latter method has been used with increasing frequency in the epidemiological and medical literature in recent years [6].

In an IPTW analysis in a setting with a binary point-exposure applied at baseline, subjects are weighted by the inverse of the probability of receiving the treatment that was actually received (as estimated using the propensity score). In this synthetic, weighted sample, treatment assignment is not confounded with measured baseline covariates if the propensity score model has been specified correctly [4, 7]. Therefore, the effect of treatment can be estimated by comparing outcomes directly between treatment groups, similar to the analyses that would be conducted in an RCT. Thus, the analysis conducted in the weighted sample can often replicate the analysis that would be conducted in an RCT if all potential confounders were considered in the propensity score model.

A marginal treatment effect refers to the difference in average outcomes between two populations, such that the only systematic difference between the two populations is that the treatment was applied to all subjects in the first population and withheld from all subjects in the second population. Alternatively, the marginal effect can be thought of as the change in average outcome, at the population level, of moving an entire population from control to treatment conditions. Marginal effects can be contrasted with conditional effects, which are the average effect of treatment at the individual level [8, 9]. From the definition of marginal effects, it is readily apparent that RCTs permit the estimation of marginal treatment effects. Due to the use of randomization, the treated and control arms are not expected to systematically differ from one another in baseline characteristics. Similarly, an IPTW analysis allows for estimation of marginal effects: the use of weights results in a synthetic sample in which treatment assignment is not confounded with measured baseline covariates. Thus, one is comparing outcomes between two populations in which measured systematic differences between treatment and control groups have been eliminated. Accordingly, a strength of an IPTW analysis is that it permits estimation of the marginal effect, which is of primary interest in RCTs. This is in contrast to conventional regression adjustment, in which one is estimating a conditional effect, which is of secondary interest in RCTs.

An important issue in designing RCTs and observational studies is the statistical power of the study design. Statistical power is the probability of detecting, as statistical significant, a true non-null treatment effect. An assessment of statistical power prior to conducting a study is important for several reasons. First, it allows the investigator to assess whether the expenditure of resources is warranted given the likelihood of detecting a clinically-meaningful effect size as statistically significant. Second, it provides both the investigator and readers with information to help interpret potentially null conclusions once the study has been completed. Conducting statistical power and sample size calculations is a routine aspect of the design of RCTs. Furthermore, methods for determining power and sample size in conventional RCTs have been well described and can be conducted easily [10, 11]. Methods for estimating statistical power have been described for observational cohort designs and case–control studies [12]. However, these methods are often overly simple or require information that may not be readily available to study investigators (e.g. correlations between the primary exposure variable and the other study covariates). Furthermore, these methods are designed for use with conditional effects (i.e. estimates obtained from adjusted regression models), rather than for use with marginal effects. It is our subjective assessment that statistical power calculations are presented less frequently in the reports of observational studies than they are in the reports of RCTs. Furthermore, sample size and power calculations can be much more difficult in observational studies that use IPTW using the propensity score. In such studies, the weights are functions of the observed data, and are not known prior to conducting the study analyses. Thus, estimates of standard errors that require knowledge of these weights cannot be obtained prior to conducting the study. Similarly, for other analyses (e.g., Cox regression in the weighted sample), closed-form expressions for the standard errors do not exist. Thus, when conducting an IPTW analysis, important quantities that are necessary to estimate statistical power are unavailable prior to the analysis being conducted. It is unknown whether the statistical power of an observational study using an IPTW analysis can be approximated by the statistical power of a similarly-structured RCT.

The objective of the current study was to compare the statistical power to detect a non-null hazard ratio in an observational study that used an IPTW analysis with the statistical power to detect a non-null hazard ratio in a similarly-structured RCT with the same number of observed events. This is an important issue as it will allow investigators designing observational studies to decide whether they can use the estimate of statistical power from a comparable RCT as an approximation to the statistical power in an observational study. Since absence of non-compliance in RCTs is a necessary condition in order to interpret effect estimates as marginal effects, we consider in the following the ideal case of RCTs without non-compliance. In particular, 100 % compliance (in all treatment arms) is a necessary condition to interpret effect estimates based on RCTs as marginal effects. Furthermore, we restrict our attention to the simplest case of a point-exposure RCT (i.e., an RCT in which exposure is applied and fixed at baseline). Monte Carlo simulations were used to obtain empirical estimates of statistical power in each of the two study designs. The paper is structured as follows: In Section 2, we describe the extensive set of Monte Carlo simulations that were used to obtain empirical estimates of statistical power. In Section 3, we report the results of these simulations. Finally, in Section 4, we summarize our findings and place them in the context of the existing literature.

## Methods

We used an extensive series of Monte Carlo simulations to compare the statistical power of an analysis of observational data that used IPTW using the propensity score with the statistical power of an RCT that had an equal number of subjects. The focus of the current simulations was on studies with a survival or time-to-event outcome, as these occur frequently in the medical literature [13]. In the subsequent two sub-sections, we describe how data were simulated to replicate an observational study and to replicate a randomized study.

### Simulating observational study data

The design of our Monte Carlo simulations was based on a recently-published study that used Monte Carlo simulations to compare the performance of different propensity score methods for estimating marginal hazard ratios [14]. The simulations in the current study were designed to examine the impact of the following four factors on the statistical power of an IPTW analysis: (i) the number of observed events; (ii) the magnitude of the true marginal hazard ratio; (iii) the proportion of subjects who were exposed to the treatment (i.e. prevalence of treatment/exposure); and (iv) the strength of the treatment-selection process (i.e. the degree of confounding). The strength of the treatment-selection process was quantified using the c-statistic (equivalent to the area under the receiver operating characteristic (ROC) curve) of the treatment-selection model. We allowed the number of observed events to take on the following values: 200 to 1000 in increments of 100, then 1000 to 5000 in increments of 1000; the marginal hazard ratio took on the following values: 1.10, 1.25, and 1.50; the prevalence of treatment took on the following values: 10, 25 and 50 %; finally the c-statistic of the treatment-selection model took on five values: 0.5, 0.6, 0.7, 0.8, and 0.9. Our simulations used a full factorial design. We thus considered 585 = 13 × 3 × 3 × 5 different scenarios.

For a given scenario, as in the prior Monte Carlo simulations, we simulated 10 baseline covariates for each of N subjects from independent standard normal distributions [14]. Of these ten covariates, seven affected treatment selection (X_1_ - X_7_), while seven affected the outcome (X_4_ - X_10_). For each subject, the probability of treatment selection was determined from the following logistic model:

1 $$ \mathrm{logit}\left({p}_i\right)={\alpha}_{0,\mathrm{treat}}+{\alpha}_W{x}_1+{\alpha}_M{x}_2+{\alpha}_S{x}_3-{\alpha}_W{x}_4+{\alpha}_M{x}_5-{\alpha}_S{x}_6+{\alpha}_{\mathrm{AUC}}{x}_7 $$

The strength of the treatment-selection process was measured using the c-statistic, which measures the degree to which the model separates or discriminates between treated and control subjects. When the c-statistic of the treatment-selection model was chosen to be 0.5, all of the regression coefficients were set to zero (i.e. none of the baseline covariates affected treatment selection). When the c-statistic of the treatment-selection model was chosen to be greater than 0.5, the regression coefficients α_W_, α_M_, and α_S_ were set to log(1.05), log(1.10), and log(1.25) respectively. These were intended to denote weak, moderate, and strong treatment-assignment affects. The final regression coefficient, α_AUC_, was chosen so that the treatment-selection model would have a specified c-statistic. The value of α_AUC_ was selected based on previously published results that relate the c-statistic of a univariate logistic regression model to the variance of the predictor variable and the odds ratio relating the predictor variable to the outcome [15]: ${\alpha}_{\mathrm{AUC}}=\sqrt{2{\left(\Phi \left(\mathrm{A}\mathrm{U}\mathrm{C}\right)\right)}^2-{\alpha}_W^2-{\alpha}_W^2-{\alpha}_M^2-{\alpha}_M^2-{\alpha}_S^2-{\alpha}_S^2}$, where Φ() denotes the standard normal quantile function and AUC denotes the desired c-statistic of the treatment-selection model. The intercept of the treatment-selection model (α_0,treat_) was selected so that the proportion of subjects in the simulated sample that were treated was fixed at the desired proportion (0.10 vs. 0.25 vs. 0.50). The value of α_0,treat_ ranged from −3.23 to 0.002, with a median of −1.22. For each subject, treatment status (Z) was generated from a Bernoulli distribution with subject-specific parameter *p*_*i*_: Z ~ Be(*p*_*i*_).

We then generated a time-to-event outcome for each subject using a data-generating process for time-to-event outcomes described by Bender et al. [16]. For each subject, the linear predictor was defined as

2 $$ \mathrm{L}\mathrm{P}={\beta}_{\mathrm{treat}}Z+{\beta}_W{x}_4+{\beta}_M{x}_5+{\beta}_S{x}_6+{\beta}_{VS}{x}_7+{\beta}_W{x}_8+{\beta}_M{x}_9+{\beta}_S{x}_{10} $$

The regression coefficients β_W_, β_M_, β_S_, and β_VS_ were set to log(1.25), log(1.5), log(1.75) and log(2), respectively. These were intended to denote weak, moderate, strong, and very strong effects on the hazard of the outcome. Note that there were two covariates (X_4_ and X_6_) that had a negative effect on treatment selection and a positive effect on outcomes. This was done to reflect settings with a treatment-risk paradox, in which higher risk patients are less likely to receive treatment [17]. The regression coefficient *β*_treat_ was set equal to 0.164156, 0.3945684, and 0.721035, to induce a marginal hazard ratio of 1.1, 1.25, and 1.5, respectively. For each subject, we generated a random number from a standard Uniform distribution: *u* ~ U(0,1). A survival or event time was generated for each subjects as follows: ${\left(\frac{- \log (u)}{\lambda {e}^{\mathrm{LP}}}\right)}^{{}^{1/\eta }}$. We set λ and η to be equal to 0.00002 and 2, respectively. The use of this data-generating process results in a conditional treatment effect, with a conditional hazard ratio of exp(β_treat_). However, we wanted to generate data in which there was a specified marginal hazard ratio (since propensity score methods and RCTs permit estimation of marginal, rather than conditional effects). To do so, we modified a previously described data-generating processes for generating data with a specified marginal odds ratio or risk difference [18, 19]. We used an iterative process that is described in greater detail elsewhere, to determine the value of β_treat_ (the conditional log-hazard ratio) that induced the desired marginal hazard ratio [14]. This process was used as we were unaware of a formula that relates the marginal hazard ratio to the conditional hazard ratio for treatment, characteristics of the distribution of the covariates in the population, and the hazard ratios relating the covariates to the hazard of the occurrence of the outcome.

Once a simulated dataset had been created, we estimated the propensity score using a logistic regression model to regress the indicator variable denoting treatment status on the seven variables that affect the hazard of the outcome (X_4_ - X_10_),. We used this set of seven variables, rather than the variables that affect treatment assignment (X_1_ – X_7_), as using the predictors of the outcome has been shown to result in superior inferences [20, 21]. The conventional inverse probability of treatment weights (IPTWs) are defined as $\frac{Z}{e}+\frac{1-Z}{1-e}$ [22], where *e* denotes the propensity score and Z denotes treatment assignment (Z = 1 treated vs. Z = 0 control). Instead of using the conventional IPTWs, we used stabilized weights, which are defined as $\frac{Z\times \Pr \left(Z=1\right)}{e}+\frac{\left(1-Z\right)\times \Pr \left(Z=0\right)}{1-e}$ [23, 24], as these weights are less susceptible to extreme weights. The quantities Pr(Z = 1) and Pr(Z = 0) denote the marginal probabilities of receiving the active treatment and the control treatment in the sample. In the simulations, the true value of the propensity score was replaced by its sample estimate, *ê* (it has been shown that using the estimated propensity score performs better than using the true propensity score [4, 25]).

In the weighted sample, we used a Cox regression model to regress survival on an indicator variable denoting treatment status and used a robust variance estimator [26, 27]. The statistical significance of the null hypothesis test for the treatment effect was derived from the fitted Cox regression model. This process was repeated 1000 times for each of the 585 scenarios. The empirical estimate of the statistical power to detect a non-null marginal hazard ratio was the proportion of simulated datasets with a true non-null hazard ratio, in which the statistical significance of the estimated hazard ratio was less than or equal to 0.05 (i.e. the proportion of simulated datasets in which the estimated marginal hazard ratio was statistically significantly different from the null with a p-value of less than or equal to 0.05).

All simulations and statistical analyses were conducted using the R statistical programming language (version 3.1.2) (The R Foundation for Statistical Computing, Vienna, Austria) including the coxph function in the ‘survival’ package (version 2.38.2).

### Simulating RCT data

The statistical power to detect a non-null marginal hazards ratio using IPTW in an observational study was compared with the statistical power to detect a non-null hazards ratio in an RCT with the same number of observed events and similar characteristics. As above, we allowed the following factors to vary: the sample size of the RCT, the prevalence of treatment (P_treat_ - the proportion of subjects randomly assigned to the active treatment arm of the RCT), and the magnitude of the effect of treatment on the hazard of the outcome (*β*_treat_ - the marginal log-hazard ratio).

For each of N subjects, we simulated ten baseline covariates as described in Section 2.1. We then simulated a treatment status from a Bernoulli distribution with parameter P_treat_: treatment was assigned at random and was not influenced by the baseline covariates. We then generated a time-to-event outcome for each subject using expression (2) and the methods described in Section 2.1. For each subject, we generated a random number from a standard Uniform distribution: *u* ~ U(0,1). A survival time was generated for each subject as follows: ${\left(\frac{- \log (u)}{\lambda {e}^{\mathrm{LP}}}\right)}^{{}^{1/\eta }}$. As above, λ and η were set to be equal to 0.00002 and 2, respectively. Thus, as above, survival is affected by both treatment and a subset of the baseline covariates. However, treatment assignment was at random, and was not affected by baseline covariates.

Once a simulated dataset had been constructed, a univariate Cox proportional hazards regression model was used to regress survival time on an indicator variable denoting treatment status. The statistical significance of the estimated hazard ratio was estimated using the model-based standard errors from the fitted Cox model. This process was repeated 1000 times for each scenario. As above, the empirical estimate of statistical power was estimated as the proportion of simulated datasets in which the estimated log-hazard ratio was statistically significantly different from zero (with a significance level of less than 0.05).

We report the empirical estimates of statistical power for the RCT design so that our methods are consistent with those used in estimating the power of an IPTW analysis. However, for RCTs, explicit formulas exist to estimate statistical power when using a Cox proportional hazards model to estimate the effect of treatment on the hazard of an outcome [11]. We compared our empirical estimates of statistical power in RCTs with the theoretical derivations provided by Schoenfeld [11]. The empirical estimates and the theoretical derivations were virtually identical across the large majority of scenarios (data not shown).

Finally, we would note that one of the factors in our Monte Carlo simulations is the number of observed events, rather than the sample size. The reason for this choice is that statistical power in survival analysis in general is related to the number of observed events, rather than to the total sample size [11]. Due to our simulating data in which subjects were not subject to censoring (i.e., subjects were followed until the event was observed to occur for all subjects), the number of observed events is equal to the number of subjects in the simulated sample.

## Results

To provide an understanding of the degree of confounding induced by the different treatment-selection models, we computed the relative bias in the crude estimate of the marginal hazard ratio in the observational data in each of the 9 scenarios with 5000 observed events. The minimum, median, and maximum relative biases are reported in Table 1. The magnitude of the bias in the estimated crude hazard ratio in the observational data increased with the c-statistic of the treatment-selection model.

**Table 1:** Relative bias in crude marginal hazard ratio

c-statistic	Minimum relative bias (%)	Median relative bias (%)	Maximum relative bias (%)
0.5	−0.7	0.4	0.6
0.6	1.9	2.7	2.9
0.7	−23.8	−20.4	−18.7
0.8	−47.4	−39.1	−35.1
0.9	−75.6	−59.6	−51.6

**Fig. 1:**
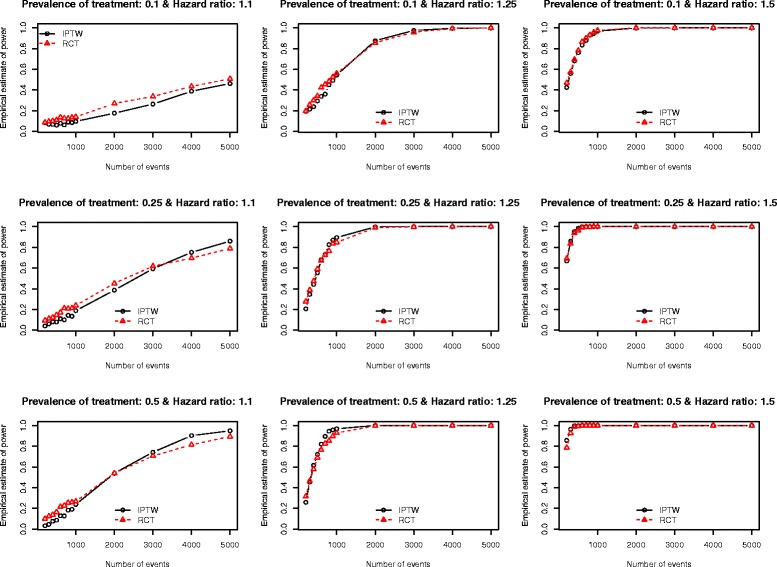
Empirical estimates of power (AUC = 0.5)

**Fig. 2:**
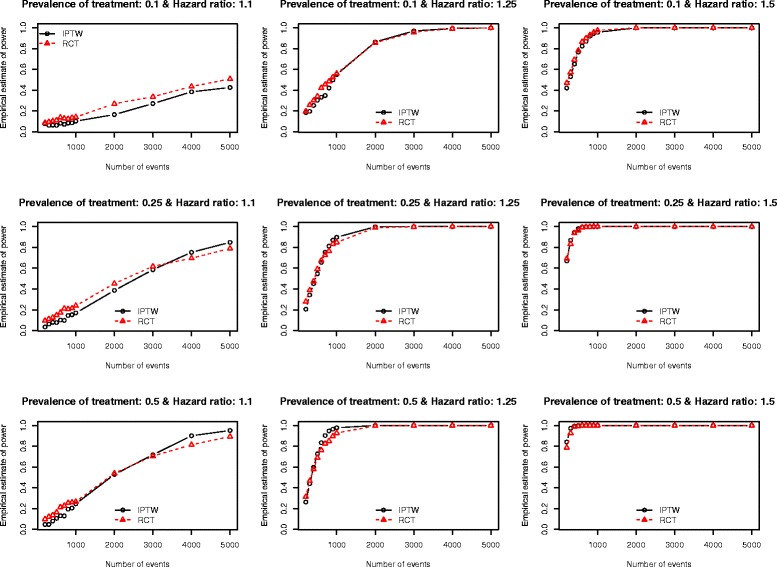
Empirical estimates of power (AUC = 0.6)

**Fig. 3:**
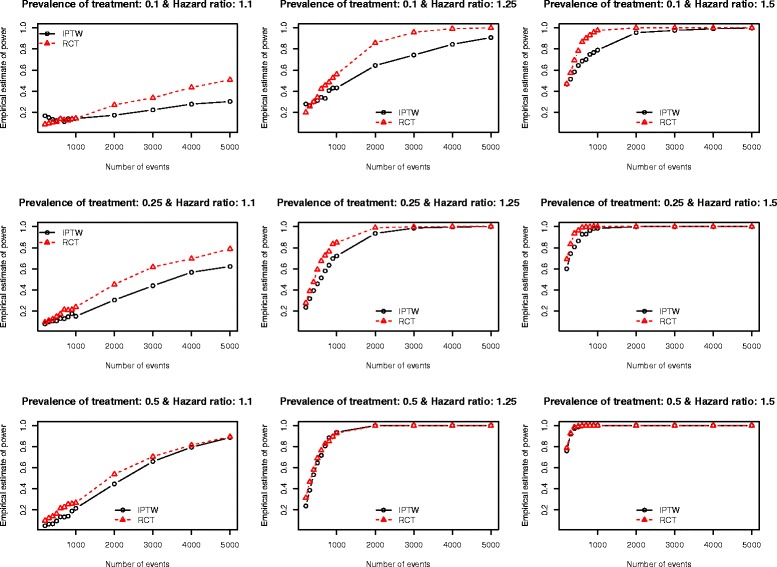
Empirical estimates of power (AUC = 0.7)

**Fig. 4:**
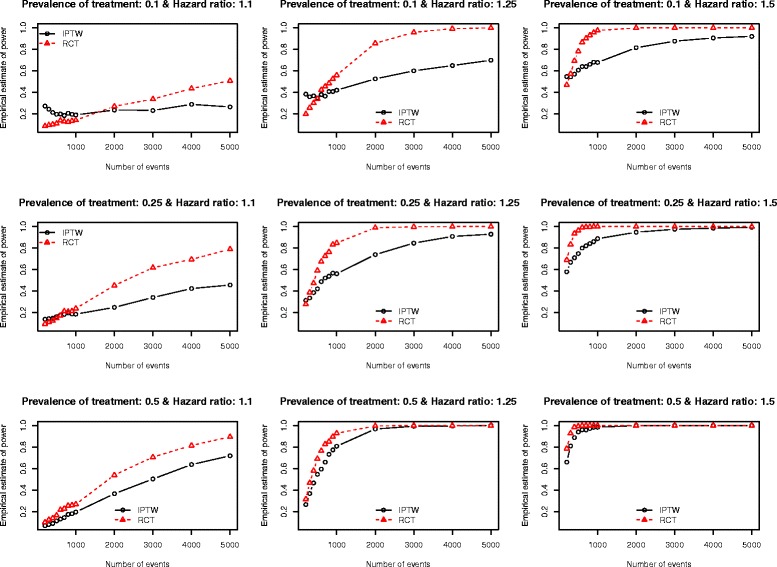
Empirical estimates of power (AUC = 0.8)

**Fig. 5:**
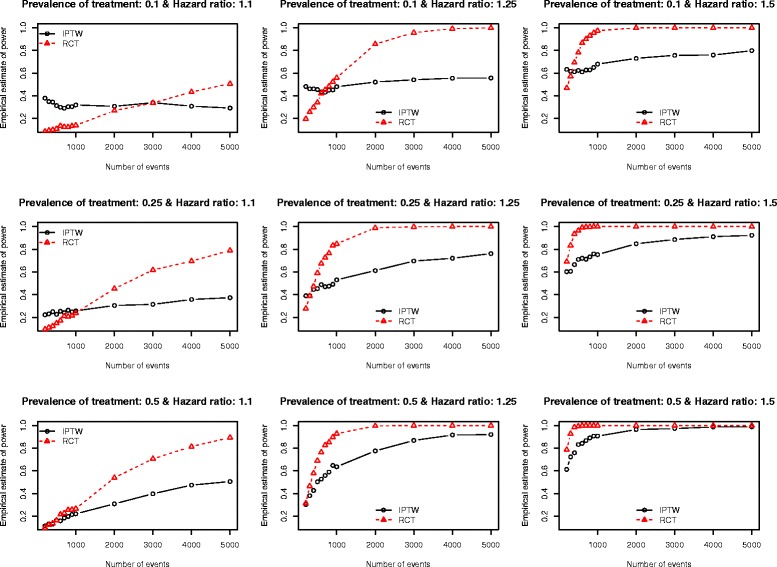
Empirical estimates of power (AUC = 0.9)

The Monte Carlo estimates of statistical power of an analysis of observational data using IPTW and the statistical power of an RCT are reported in Figs. 1, 2, 3, 4 and 5. There is one figure for each of the five different c-statistics of the treatment-selection model (0.5, 0.6, 0.7, 0.8, and 0.9). Within each of the five figures there is one panel for each of the nine combinations of the true marginal hazard ratio (1.1 vs. 1.25 vs. 1.5) and the prevalence of treatment (10 % vs. 25 % vs. 50 %). Several findings merit comment.

First, when focused on RCTs, statistical power increased with increasing number of events, with increasing underlying hazard ratio, and with increasing prevalence of treatment. Of these three factors, the latter factor had the smallest impact on statistical power. None of these observations are surprising. We highlight these observations primarily to provide context for subsequent findings and observations.

Second, the results from the setting of an observational study in which treatment-selection was random (i.e. the c-statistic of the treatment-selection model was 0.5, indicating an absence of confounding because none of the covariates influenced treatment selection) are reported in Fig. 1. In this setting, one observes that the use of IPTW in an observational study gives slightly lower statistical power than an RCT in the scenario with both a low prevalence of treatment (10 %) and a low effect size (hazard ratio = 1.1). When the hazard ratio was moderate (1.25) or large (1.5), then the two designs had approximately equivalent statistical power. When the hazard ratio was low (1.1) and treatment prevalence was moderate or high (25 % or 50 %), then an RCT design had slightly higher statistical power when the number of events was less than 2000 or 3000. An important conclusion to draw from these results is that, in most settings, in the absence of confounding, the use of an IPTW analysis (instead of a crude or unadjusted analysis) does not result in a meaningful decrease in statistical power.

Third, for a fixed treatment prevalence and underlying marginal hazard ratio, the differences in statistical power between RCTs and observational studies tended to increase as the strength of the treatment-selection process increased (i.e. with increasing c-statistic of the treatment-selection model).

Fourth, as noted above, when there was no confounding, an RCT design tended to have an equal, or marginally greater, statistical power than an observational design analyzed using IPTW. However, as the degree of confounding increased (as measured using the c-statistic of the treatment-selection process), the number of scenarios in which an IPTW analysis had greater statistical power than the RCT tended to increase. This inversion in statistical power was evident primarily at lower sample sizes. The sample sizes for which this inversion existed increased as the degree of confounding increased. Scenarios in which the IPTW analysis had greater statistical power than the RCT analysis tended to be restricted to scenarios in which the true marginal hazard ratio was low (1.1) or moderate (1.25). When the true hazard ratio was large (1.5), then the RCT tended to have statistical power that was at least as great as that of the observational design.

**Fig. 6:**
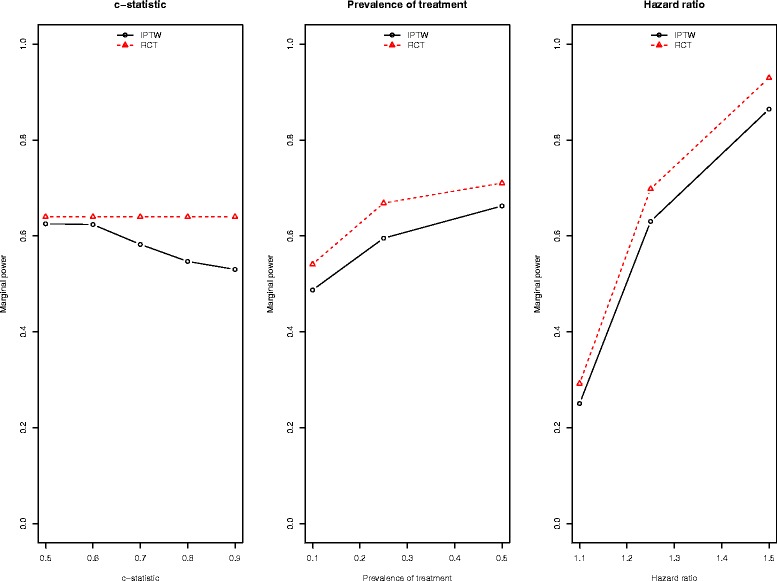
Marginal estimates of empirical power

In Fig. 6 we report marginal (or average) estimates of statistical power across the different values of each of three factors (c-statistic, prevalence of treatment, and marginal hazard ratio). There is one panel for each of these three factors. Each panel reports the marginal (or average) estimate of statistical power for an IPTW analysis and for an RCT design. In examining marginal estimates of statistical power, we note the following: (i) the average estimate of power was greater for the RCT design than for the IPTW analysis; (ii) differences in power between the two designs were amplified as the strength of the treatment-selection model increased (left panel); (iii) average estimates of power for each design increased with increasing prevalence of treatment (middle panel) and with increasing hazard ratio (right panel). The latter observation is unsurprising, and the focus should be on the first two observations.

## Discussion

We conducted an extensive series of Monte Carlo simulations to compare the statistical power to detect a non-null hazard ratio using IPTW using the propensity score in an observational study with the statistical power to detect a non-null hazard ratio in an RCT of the sample size. The primary motivation of these simulations was to provide applied researchers using observational data to estimate treatment effects with insight into the statistical power of their analyses. In particular, we were interested in whether the statistical power of an RCT, which can be easily estimated, provides a reasonable approximation to the statistical power of an observational study analyzed using IPTW. This is an important question, as the power of an RCT can be readily estimated prior to the implementation of the trial. In contrast, an IPTW analysis requires the use of the IPT weights, which can only be estimated once the data have been collected. Thus, it is not readily feasible to estimate directly the power of an observational study employing IPTW prior to the data being collected. We sought to determine whether the power of a similarly-structured RCT can provide an adequate approximation to the power of an observational study that employs IPTW.

Stürmer et al. report on a systematic review that examined articles published in the medical literature between 1997 and 2003 that used propensity score methods [28]. Seventy-three articles reported the exact c-statistic of the propensity-score model. The 25^th^, 50^th^, and 75^th^ percentiles of the reported c-statistics were 0.71, 0.80, and 0.84, respectively. The minimum and maximum reported c-statistics were 0.56 and 0.94, respectively. Thus, the large majority of published observational studies had reported c-statistics that fell within the range of c-statistics that we examined in our simulations. Furthermore, half of the published studies had c-statistics that fell between 0.71 and 0.80. When examining our findings when the c-statistic was 0.7 (Fig. 3) and 0.8 (Fig. 4), we make some additional observations. First, when the true marginal hazard ratio was low and the c-statistic was 0.7, then the power of the IPTW analysis tended to be less than that of the RCT design. Second, when the c-statistic was 0.8 and the marginal hazard ratio was low to moderate, then the power of the IPTW analysis tended to be less than that of the RCT design. In some cases, the difference in statistical power was substantial. Thus, in settings typical to that seen in many observational studies in the medical literature (i.e. c-statistics of 0.7 or 0.8), the statistical power of an RCT with equivalent sample size may not provide a good approximation of the statistical power of an observational analysis using IPTW. In order to appreciate the degree of confounding associated with c-statistics of these magnitudes, we refer the reader to Table 1 of the current paper. In our simulations, a c-statistic of 0.7 was associated with a relative bias in the estimated crude hazard ratio of between −23.8 % and −18.7 %, while a c-statistic of 0.8 was associated with a relative bias in the crude hazard ratio of between −47.4 % and −35.1 %.

In some settings in which there was a very strong treatment-selection process (i.e., a very high c-statistic for the treatment-selection model), we observed that the IPTW design had greater statistical power than the RCT design. This was evident particularly when the number of events was low and the prevalence of treatment was low to moderate. There are several possible explanations for this somewhat surprising observation. First, it is possible that in some iterations of the Monte Carlo simulations, large stabilized weights resulted in inflated estimates of the regression coefficient, leading to rejection of the null hypothesis. Second, one observes that some of these power curves are not monotone increasing (e.g., top left panel of Fig. 5). This may indicate that the standard error estimates for the IPTW analysis are too liberal (for potentially inflated effect estimates), at least for a lower numbers of events. This issue requires further exploration in subsequent research. Third, Rosenbaum has stated that using the estimated propensity scores induces better balance in measured baseline covariates compared to when the true propensity scores is used [4]. It is possible that this effect is more pronounced in the settings with a low number of events and that it resulted in an artificially high statistical power for the IPTW design. Fourth, in a set of exploratory analyses, we examined the empirical type I error rates of the two designs in a set of scenarios in which there was a true null treatment effect. As expected, the RCT design tended to have empirical type I error rates that were not statistically significantly different from the advertised rate of 0.05. However, the empirical type I error rate of the IPTW design was often significantly different from the advertised rate of 0.05 (data not shown). If the IPTW design does not, in some settings, maintain the anticipated alpha level, this can be an issue when comparing power curves, because such comparisons require the same alpha levels of the estimators being compared. This issue requires further exploration in subsequent research.

There are certain limitations to the current study that warrant mention. First, we acknowledge that RCTs are considered the gold standard for estimating the effects of treatments and interventions since treatment assignment is not confounded with subject characteristics. We are not suggesting that observational studies are never subject to unmeasured confounding. Our primary objective was to compare the statistical power of observational studies in which there is no *unmeasured* confounding with the statistical power of RCTs. Depending on the nature of the data used in the observational study, unmeasured confounding may be an issue [29]. If the assumption of no unmeasured confounding does not hold, however, any inference from an observational study is invalid, and the issue of power should not be relevant. Second, our analyses relied upon Monte Carlo simulations due to the inability to derive closed-form expression for the statistical power of analyses that use IPTW using the propensity score with time-to-event outcomes. Due to our use of simulations, we were only able to examine a limited number of scenarios. However, we did examine 585 scenarios that reflected a wide range of scenarios, and that included hazard ratios that reflect meaningful effect sizes in the medical literature. Furthermore, by allowing the discrimination of the treatment-selection model vary from 0.5 to 0.9, we considered situations with a wide range of confounding, reflective of scenarios encountered in observational research [28]. Third, we want to emphasize that our simulation studies considering time-to-event outcomes did not incorporate censored observations. However, since statistical power in survival analysis relies on the number of observed events and not directly on overall sample sizes, our results should be readily generalizable to common settings of RCTs and observational studies including censored data. We would highlight that our objective was not to determine the statistical power of each method in isolation. Instead, it was to compare the statistical power of an IPTW design with that of a similarly-structured RCT. We can think of no rationale for why the effect of censoring on statistical power would differ between the two designs. Fourth, we used the c-statistic to quantify the strength of the treatment-selection process. However, the c-statistic does not take the number of model covariates into account. Thus, we did not examine the sensitivity of the power of the IPTW approach to the number of covariates in the treatment-selection model. However, the current study examined 585 scenarios, and it would have been computationally burdensome to expand the simulations to add an additional factor, the number of covariates in the treatment-selection model. However, this merits examination in a subsequent study.

## Conclusion

Conducting an a priori power calculation for an observational study that uses IPTW is difficult since such a calculation would rely on the IPT weights, which are only known after the analysis has been conducted. It would be attractive to be able to use the power of similarly-structured RCT as an approximation of the power of an IPTW analysis. However, analyses of observational data with time-to-event outcomes using IPTW methods had, on average, lower statistical power than did analyses of similarly-structured RCTs. The magnitude of the difference in statistical power increased as the strength of the treatment-selection model increased. The statistical power of an RCT does not necessarily provide an accurate estimate of that for an IPTW analysis.

## Abbreviations

IPTW: Inverse probability of treatment weighting
IPT: Inverse probability of treatment
RCT: Randomized controlled trial
